# Deaths from Chloroform in New South Wales

**Published:** 1890-11

**Authors:** 


### DEATHS FROM CHLOROFORM IN NEW SOUTH WALES.

Through the kindness of Dr. Alfred Burne, of Sydney, Australia, we are in receipt of slips giving a report of a discussion in the journals of that city relative to the alarming increase in deaths by chloroform which have occurred in New South Wales since 1885, and especial attention is called to the fact that in Prince Alfred Hospital three deaths have taken place in a "few weeks." These communications have more than a local value but are too extended for our space. The exhibit will, doubtless, be read with interest by those who regard the A.-C.-E. mixture entirely harmless.

